# Laparoscopy for emergency abdominal surgery is associated with reduced physical functional decline in older patients: a cohort study

**DOI:** 10.1186/s12877-024-04872-y

**Published:** 2024-03-12

**Authors:** Keishi Yamaguchi, Takeru Abe, Shokei Matsumoto, Kento Nakajima, Masayuki Shimizu, Ichiro Takeuchi

**Affiliations:** 1 Department of Emergency Medicine, Yokohama City University Graduate School of Medicine, 4-57 Urafunecho, Minamiku, Yokohama, 232-0024 Japan; 2 Department of Trauma and Emergency Surgery, Saiseikai Yokohamashi Tobu Hospital, Yokohama, Japan; 3 Center for Integrated Science and Humanities, Fukushima Medical University, Fukushima, Japan

**Keywords:** Acute care surgery, Barthel index, Emergency abdominal surgery, Geriatric surgery, Laparoscopic surgery, Older patient, Physical function, Quality of life

## Abstract

**Background:**

An increasing number of older patients require emergency abdominal surgery for acute abdomen. They are susceptible to surgical stress and lose their independence in performing daily activities. Laparoscopic surgery is associated with faster recovery, less postoperative pain, and shorter hospital stay. However, few studies have examined the relationship between laparoscopic surgery and physical functional decline. Thus, we aimed to examine the relationship between changes in physical function and the surgical procedure.

**Methods:**

In this was a single-center, retrospective cohort study, we enrolled patients who were aged ≥ 65 years and underwent emergency abdominal surgery for acute abdomen between January 1, 2019, and December 31, 2021. We assessed their activities of daily living using the Barthel Index. Functional decline was defined as a decrease of ≥ 20 points in Barthel Index at 28 days postoperatively, compared with the preoperative value. We evaluated an association between functional decline and surgical procedures among older patients, using multiple logistic regression analysis.

**Results:**

During the study period, 852 patients underwent emergency abdominal surgery. Among these, 280 patients were eligible for the analysis. Among them, 94 underwent laparoscopic surgery, while 186 underwent open surgery. Patients who underwent laparoscopic surgery showed a less functional decline at 28 days postoperatively (6 vs. 49, *p* < 0.001). After adjustments for other covariates, laparoscopic surgery was an independent preventive factor for postoperative functional decline (OR, 0.22; 95% CI, 0.05–0.83; *p* < 0.05).

**Conclusions:**

In emergency abdominal surgery, laparoscopic surgery reduces postoperative physical functional decline in older patients. Widespread use of laparoscopic surgery can potentially preserve patient quality of life and may be important for the better development of emergency abdominal surgery.

**Supplementary Information:**

The online version contains supplementary material available at 10.1186/s12877-024-04872-y.

## Background

The global older population, aged ≥ 65 years, is estimated to increase to > 1.5 billion by 2050 from 524 million in 2010 [1]. With the rapid aging of the population, acute abdomen has become a global challenge, as the number of older people requiring emergency abdominal surgery is increasing annually [2–4]. Owing to surgery, older patients are more likely to experience loss of autonomy in daily living, in addition to increased postoperative complications, mortality, and resource utilization [5, 6]. The loss of autonomy burdens the patients, their families, and society [7]. Additionally, it is an independent predictor of re-hospitalization and death after discharging patients from the hospital [8]. For older individuals, outcomes such as reduced independence and quality of life (QOL) are more crucial than mortality itself [9], and ≥ 70% of older individuals decline any treatment that would cause severe functional impairment due to reduced QOL, even if survival is guaranteed [5].

In recent years, the usefulness of laparoscopic surgery (LS) in older patients requiring emergency surgery has been reported [1, 10], and it is being widely used as an elective surgery among them. The advantages of LS include reduced surgical site infections (SSIs) [11–13], less postoperative pain, a faster recovery, shorter hospital stays, a faster return to work and resumption of daily activities, and cosmetic advantages [14, 15]. Despite these advantages, many patients undergo open surgery (OS) for various reasons during emergency surgeries, which may be surgeon-specific, such as diffuse peritonitis or technical difficulties associated with adhesions, or patient-specific, such as shock vitals [16, 17]. Consequently, there is no clear consensus on laparoscopic emergency abdominal surgery, excluding appendectomy and cholecystectomy [14, 17, 18]. Nevertheless, the potential benefits of LS for acute abdomen have been highlighted [19] and found to be associated with reductions in the duration of hospital stay [20] and lower postoperative mortality rates in older individuals [1]. However, few studies have examined the relationship between LS and physical functional decline.

Hence, we hypothesized that the advantages of LS, including less postoperative pain and a faster recovery, would contribute to the prevention of postoperative functional decline in older patients. In this study, we aimed to assess the preoperative and postoperative physical function of older patients who underwent emergency abdominal surgery and examine the relationship between changes in physical function and the surgical procedures.

## Methods

### Study design

This was a single-center, retrospective cohort study of patients who underwent emergency abdominal surgery between January 1, 2019 and December 31, 2021 at the Emergency and Trauma Center of Saiseikai Yokohamashi Tobu Hospital, a tertiary-care hospital in Yokohama, Japan. We included patients aged ≥ 65 years who underwent emergency abdominal surgery for acute abdomen. Patients undergoing vascular or gynecological surgery, trauma surgery, patients with complications from elective surgery, and those who died during hospitalization were excluded.

### Ethical considerations

The study protocol was approved by the Institutional Review Board, the Ethics Committee of the Saiseikai Yokohamashi Tobu Hospital (No. 20210173) and informed consent was waived by the Institutional Review Board, the Ethics Committee of the Saiseikai Yokohamashi Tobu Hospital in Japan. The individual informed consent was opted out, due to a nature of retrospective study design, per Chapter 1, Article 2, Section 5 in the Personal Information Protection Law and the National Research Ethics Guideline in Japan. This study was conducted in accordance with the principles of the Declaration of Helsinki, and has been reported in line with the STROBE statement [21].

### Clinical procedures

A surgeon directly examined patients presenting with acute abdominal pain to determine the need for emergency abdominal surgery. OS was considered for any disease if the patient’s vital signs were unstable. The surgeon chose OS or LS in patients with stable vital signs, based on each surgeon’s discretion. The procedure was converted to OS if LS was infeasible during the surgery. LS used multiport and single-port incisions to establish pneumoperitoneum and facilitate surgical resection. In LS, median incisions were used only to facilitate specimen collection. OS was performed using a standard median incision. Postoperatively, patients were treated according to the institution’s protocol.

### Activities of Daily Living (ADLs) evaluation and functional decline

Impairment in ADLs was assessed using the Barthel Index (BI), which measures independence in mobility and personal care. The BI has been reported to be the best scale for assessing ADLs [22, 23]. An attending physician and a nurse evaluated the BI on admission and the rehabilitation team scored the BI on postoperative day 28. Patients discharged or transferred within 28 days were assessed by interviewing their family or the facility staff. The BI is a reliable and accurate measure of autonomy in ADLs and is sensitive to small changes in functional capacity [24, 25]. The BI items are related to self-care (eating, grooming, bathing, dressing, defecation/urination, and using the toilet) and mobility (walking, moving, and climbing stairs). The scale ranges from 0 for a completely dependent bedridden state to 100 for a completely independent state. Additionally, bedridden patients (a pre-morbid Barthel Index score of < 25) were excluded due to the purpose of this study, which was to evaluate the association between emergency abdominal surgery and functional decline [26]. Functional decline was defined as a decrease in BI score at 28 days postoperatively compared with the preoperative value. A decrease of ≥ 20 points in BI indicated a complete functional decline in two domains or the need for new assistance in four domains (see Additional file 1) [27]. As the patients’ QOL declines, the burden of caregiving increases; therefore, we defined this condition as functional decline [28].

In accordance with our hospital’s clinical practice program, the ERAS program was followed as closely as possible for perioperative and postoperative care. Early postoperative physical therapy began the day after surgery if the patient remained stable. For stable laparotomy patients, epidural anesthesia was employed unless contraindicated. The rehabilitation program was specifically designed for individuals, using a multidisciplinary team approach. The rehabilitation team comprised rehabilitation physicians, physiotherapists, and nurses. Rehabilitation was implemented for 30–60 min of daily activities, 5 days per week.

### Data collection

We collected the following data: age; sex; body mass index (BMI); Charlson Comorbidity Index (CCI); emergency room vital signs; blood tests such as albumin (Alb) for nutritional markers, white blood cell count (WBC) and C-reactive protein (CRP) for inflammatory markers, lactic acid for tissue ischemia markers, and total bilirubin and creatinine for other major organ markers; ADLs on admission and 28 days postoperatively; American Society of Anesthesiologists (ASA) scores; indications for emergency surgery; surgical procedure; operative time; transfusion volume; and blood loss. Surgical procedures were categorized as either OS or LS, in which the latter included conversion cases. The type of surgical intervention was categorized as either major or intermediate-minor. We defined major surgery as surgical interventions such as bowel resection, Hartmann’s surgery, or surgery for diffuse peritonitis and intermediate-minor surgery as cholecystectomy (excluding diffuse peritonitis), appendicectomy, dissection of adhesions, stoma creation without resection, hernia repair, diagnostic LS or OS [29].

### Statistical analysis

Continuous variables are expressed as medians and interquartile ranges (IQRs) and categorical variables are expressed as frequencies and percentages. We compared all study variables between the OS and attempted LS groups. The student’s t-test or Mann–Whitney test was used for continuous variables, and the chi-squared test or Fisher’s exact test was used for categorical variables. Multiple logistic regression analysis was used to examine the association between functional decline and surgical procedures—OS or LS—in older patients who underwent abdominal surgery, controlling for other factors. We included variables with *p* < 0.10 in the multiple logistic regression model with backward elimination. We included for the following as independent variables: age, sex, BMI, CCI, vital signs on admission, blood data such as pH, Alb, WBC, and hemoglobin (Hb), and surgical data such as ASA, type of surgical intervention, blood transfusion, and blood loss. Multicollinearity was defined as a variance inflation factor > 4.0, and a variable was excluded from the model when multicollinearity was observed. Similarly, after excluding cholecystitis and appendicitis, we also performed a sensitivity analysis with backward elimination. A power analysis was performed to determine the detectable percentage difference in functional decline for two patient-groups’ sample sizes. Statistical significance was set at a two-sided *p*-value of < 0.05. Odds ratios (OR) and 95% confidence intervals (CI) were also calculated. All statistical analyses were performed using the Package ‘RcmdrPlugin.EZR’ in R 4.1.1 (R Core Team (2021)). R: a language and environment for statistical computing. R Foundation for Statistical Computing, Vienna, Austria. URL: https://www.R-project.org/). The power analysis was performed with PASS 14 Power Analysis and Sample Size Software (2015) (NCSS, LLC. Kaysville, Utah, USA; ncss.com/software/pass.)

## Results

**Fig. 1:**
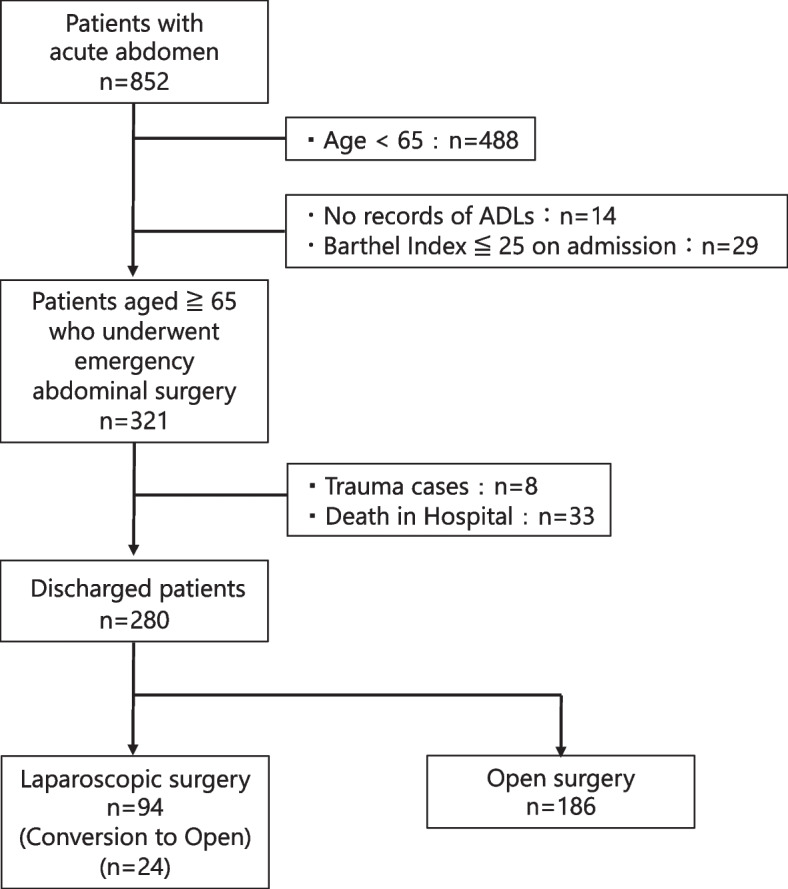
Flow diagram of patient selection. In this study, a total of 852 patients were included. Statistical analysis was performed on the data of 280 older patients, aged ≥ 65 years, who met the inclusion criteria. ADLs, Activities of Daily Living

During the study period, 852 patients underwent emergency abdominal surgery. Of these, 280 patients were eligible for the analysis (Fig. 1), among which 94 underwent LS and 186 underwent OS (Table 1).

**Table 1:** Characteristics of older patients stratified by surgical procedure

Characteristics		Laparoscopic surgery *n* = 94	Open surgery *n* = 186	*p*-value
Patient variable				
Age [years], median (IQR)		73 (70–81)	80 (73–85)	< 0.001
Female sex, n (%)		41 (43.6)	92 (49.5)	0.38
BMI: median (IQR)	kg/m^2^	22.6 (19.8–25.3)	21.3 (19.0–23.7)	0.01
Vital Signs, median (IQR)				
Respiratory Rate	/min	18 (16–20)	19 (17–23)	0.03
Heart Rate	/min	84 (72–96)	89 (75–100)	0.04
Systolic Blood Pressure	mmHg	130 (118–154)	134 (111–155)	0.81
Body Temperature	^◦^C	36.7 (36.3–37.3)	36.6 (36.3–37.0)	0.48
CCI, n, (%)				< 0.001
≥ 4		50 (53.2)	137 (73.7)	
Blood tests, median (IQR)				
pH		7.39 (7.36–7.43)	7.41 (7.37–7.44)	0.03
Lactate	mg/dL	16 (11–22)	18 (12–32)	0.16
Albumin	g/dL	4.0 (3.6–4.3)	3.5 (2.9–4.0)	< 0.001
White Blood Cell	× 1000/μL	13 (8.1–15)	9.5 (6.6–13)	< 0.01
Platelet	× 10000/μL	20.8 (18.0–26.1)	22.5 (17.1–28.5)	0.43
Hemoglobin	g/dL	13.5 (12.6–14.9)	12.3 (10.4–13.8)	< 0.001
Creatine	mg/dL	0.9 (0.7–1.1)	0.9 (0.7–1.5)	0.31
C-reactive protein	mg/dL	3.6 (0.3–13)	2.2 (0.2–13)	0.93
Bilirubin	mg/dL	0.9 (0.6–1.5)	0.8 (0.6–1.1)	0.11
ASA score, n, (%)				< 0.001
I		10 (10.6)	3 (1.6)	
II		68 (72.3)	90 (48.4)	
III		15 (16.0)	83 (44.6)	
IV		1 (1.1)	10 (5.4)	

*Abbreviations*: *IQR* interqartile range, *CCI* Charlson Comorbidity Index, *ASA* American Society of Anesthesiologists

The OS group was significantly older and had a lower BMI, higher CCI, higher ASA, faster respiratory rate, and more tachycardia (age, *p* < 0.001; BMI, *p* = 0.013; CCI, *p* < 0.001; ASA, *p* < 0.001; respiratory rate, *p* = 0.032; heart rate, *p* = 0.037) than the LS group. The details of emergency abdominal surgery are presented in Table 2.

**Table 2:** Surgical details

Characteristic		Laparoscopic surgery *n* = 94	Open Surgery *n* = 186	*p*-value
Diagnosis, n, (%)				
Appendicitis		27 (28.7)	7 (3.8)	
Cholecystitis		32 (34.0)	19 (10.2)	
Complicated hernia		4 (4.3)	26 (14.0)	
Gastric/Duodenal Perforation		4 (4.3)	7 (3.8)	
Bowel				
Small bowel adhesion		2 (2.1)	10 (5.4)	
Obstruction (cancer)		4 (4.3)	17 (9.1)	
Ischemia		16 (17.0)	48 (25.8)	
Perforation		3 (3.2)	36 (19.4)	
Other		2 (2.1)	16 (8.6)	
Time, median (IQR)	min	99.5 (65.0–136.8)	113 (80.0–154.0)	0.04
Balance (IQR)	ml			
Infusion		1075 (863–1449)	1700 (1200–2600)	< 0.001
Transfusion		0 (0–0)	0 (0–480)	< 0.001
Bleeding		0 (0–30)	71 (10–288)	< 0.001
Surgical Intervention, n, (%)				< 0.001
Major		17 (18.1)	106 (57.0)	
Intermediate-Minor		77 (81.9)	80 (43.0)	
Outcome				
Hospital LOS, (IQR)	days	8 (5–11)	15 (10–26)	< 0.001
Functional decline, n (%)		6 (6.4)	49 (26.3)	< 0.001
Complications, n (%)				
All		15 (16.0)	71 (38.2)	< 0.001
CD grade I/II		8 (8.5)	49 (26.3)	< 0.001
CD grade ≥ III		7 (7.4)	22 (11.8)	0.30
Surgical site infection (excluding intra-abdominal abscess)		5 (5.3)	27 (14.5)	0.03
Paralytic ileus		0 (0)	16 (8.6)	< 0.01
Intra-abdominal abscess		4 (4.3)	13 (7.0)	0.44

*Abbreviations*: *IQR* interqartile range, LOS length of stay, *CD* Clavien–Dindo classification

Overall, LS was converted to OS intraoperatively in 24 patients (25.5%) in the LS group. Furthermore, 82% of surgical interventions in the LS group were intermediate-minor, while 43% in the OS group were major (*p* < 0.001). The list of the type of surgical intervention in LS and OS group is also presented in Additional file 2.

**Fig. 2:**
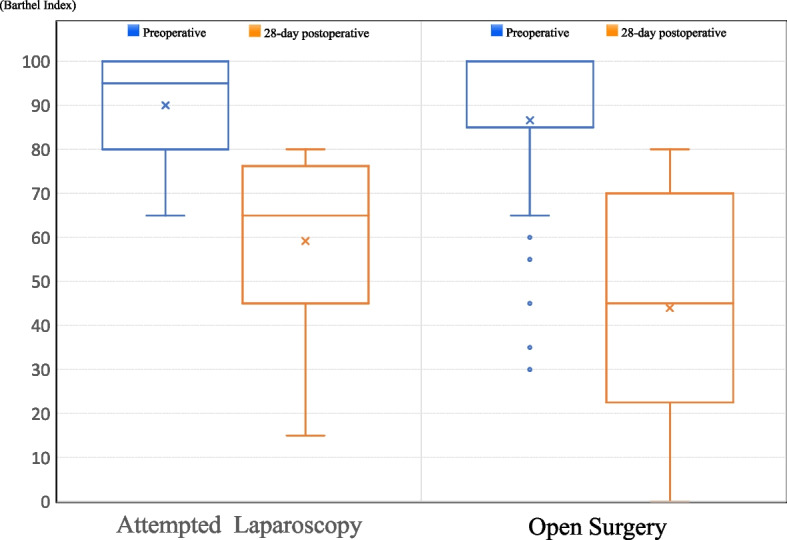
A comparison of functional decline in the two groups. The median BI declined from 95 to 65 for LS and from 100 to 45 for OS

Table 2 also shows comparisons of outcomes between the two groups. LS showed the less functional decline at 28 days postoperatively (6.4% vs. 26.3%, *p* < 0.001). Comparing patients with functional decline, the median BI in the LS group declined from 95 (IQR: 86.3–100) to 65 (IQR: 57.5–72.5). In contrast, the median BI in the OS group declined from 100 (IQR: 85–100) to 45 (IQR: 25–70). The declines in the LS and OS groups were 30 and 55, respectively, indicating a more severe functional decline with OS (Fig. 2). All postoperative complications were significantly lower in the LS group (16.0% vs. 38.2%, *p* < 0.001) than in the OS group. There was no statistical difference in LS and OS when comparing the same surgical intervention (major vs. major or intermediate minor vs. intermediate minor) (Additional file 3). Surgical site infection was also significantly less frequent in the LS than in the OS group (5.3% vs. 14.5%, *p* = 0.028).

In the multivariable logistic regression model, variables with *p* < 0.10 were included by backward elimination. The following factors were included as independent variables: age, sex, BMI, CCI, vital signs on admission, blood data such as pH, Alb, WBC, and hemoglobin (Hb), ASA, and surgical data such as type of surgical intervention, blood transfusion, and blood loss. After adjustments for covariates, age, sex, and ASA grade, surgical procedures were significantly associated (overall regression model, *p* < 0.001) with postoperative functional decline (Table 3).

**Table 3:** Association between the emergency abdominal surgery and postoperative functional decline^*^

Covariate	Odds ratio	95% Confidence Interval	*p*-value
Age per year	1.08	1.02–1.15	0.005
Male	0.38	0.16–0.90	0.027
ASA score	2.53	1.30–4.93	0.006
Surgical procedures (laparoscopic surgery)	0.22	0.05–0.83	0.025

*Abbreviations*: *ASA* American Society of Anesthesiologists

^*^Postoperative functional decline was defined as a decrease in BI score at 28 days postoperatively compared with the preoperative value. A decrease of ≥ 20 points in BI indicated a complete functional decline in two domains or the need for new assistance in four domains

LS was an independent preventive factor for postoperative functional decline (OR, 0.22; 95% CI, 0.05–0.83; *p* < 0.05). Power analysis for two-group sample size of 186 and 94 revealed that a difference in proportion of 19.9% could provide the power greater than 99% and be confidently detected as an obtained group difference.

In cases of acute appendicitis and acute cholecystitis, the laparoscopic approach is commonly chosen for surgery. By excluding these surgeries, we could get a better understanding of the benefit of LS. Thus, after excluding cholecystitis and appendicitis, a sensitivity analysis was also performed using backward elimination (Table 4).

**Table 4:** Sensitivity analysis result: Association between the emergency abdominal surgery and postoperative functional decline^*^

Covariate	Odds ratio	95% Confidence Interval	*p*-value
Age per year	1.08	1.02–1.15	0.011
Male	0.33	0.14–0.81	0.011
ASA score	2.28	1.17–4.44	0.015
Surgical procedures (laparoscopic surgery)	0.20	0.04–0.98	0.047

*Abbreviations*: *ASA* American Society of Anesthesiologists

^*^Postoperative functional decline was defined as a decrease in BI score at 28 days postoperatively compared with the preoperative value. A decrease of ≥ 20 points in BI indicated a complete functional decline in two domains or the need for new assistance in four domains

## Discussion

In this study, we examined older patients requiring emergency abdominal surgery at a general tertiary hospital to determine the evolution of the surgical approach and physical function in older patients using the BI. The most important outcomes for older patients requiring emergency surgery, apart from morbidity and mortality, are reduction in functional decline and preservation of preoperative physical function [5]. Thus, prediction of functional decline is critical; however, only a few studies have been conducted on this topic. Although this study found that chronological age is statistically associated with postoperative physical function decline, functional decline cannot be predicted using age alone [30]; frailty, defined as a decrease of physiological reserve, is a greater risk factor for functional decline than age [31]. Several tools exist to identify frailty in emergency cases, and in a simplified approach, previously, we revealed that low psoas muscle volume predicted early postoperative functional decline [32]. In addition, recent studies have shown that the use of laparoscopy in emergency abdominal surgery demonstrates a significant reduction in mortality in frail older patients [33]. This study found that LS, compared to OS, was less likely to be statistically associated with a postoperative functional decline in older individuals. We believe this analysis suggests that LS, as an alternative to OS, has a significant positive impact on the outcome of older patients after emergency abdominal surgery.

Previous studies have also shown that LS in emergency abdominal surgery reduces blood loss, length of hospital stay, and in-hospital mortality [20, 34]. These results suggest the potential to improve the quality of care more directly than factors such as hospital staffing levels, rapid access to diagnostic tools, and lack of operating room capacity, as shown in previous studies [35, 36].

One advantage of LS is its faster recovery due to smaller wounds [14, 15]. Hence, it is thought that the reduction of postoperative motion limitations due to less pain and sufficient early rehabilitation intervention will prevent postoperative functional decline. In addition, previous studies reported that LS may reduce paralytic ileus in elective surgery [37]. LS is associated with a smaller wound and less systemic inflammation, potentially leading to a faster postoperative recovery of bowel function and a lower risk of paralytic ileus [37, 38]. The results of this study are consistent with and complement these findings, indicating that our study results do not contradict existing literature and further suggest a similar potential in emergency surgeries. A previous study showed that patients who converted from LS to OS had better outcomes than those who underwent OS from the beginning [34]. In this study, patients who converted from LS to OS were included in the LS group, and all patients who underwent LS, regardless of the success or conversion of the surgical procedure, showed a reduction in blood loss, length of hospital stay, and functional decline than those who underwent OS. This may have resulted from the benefit of laparoscopic partial completion and visual confirmation of the diagnosis, allowing smaller, more targeted open incisions [34].

Indeed, it is important to recognize that these advantages are inapplicable to all emergency abdominal surgeries. Physiologically unstable patients due to hemorrhage or sepsis require emergency OS. However, LS is feasible in many cases, including small bowel obstruction by a single adhesive band, ischemic small bowel strangulation, large bowel obstruction due to colorectal cancer, diffuse peritonitis with intra-abdominal abscess, and incisional hernia [14]. Moreover, LS has revolutionized the treatment of complicated diverticulitis with bowel perforation. Laparoscopic lavage has recently emerged as an effective treatment for perforated diverticula with purulent peritonitis. Furthermore, even when Hartmann’s surgery is required, sigmoid resection can be safely performed laparoscopically, and a stoma can be formed after specimen collection [39].

In addition, as previous studies have shown [34], LS is non-inferior in terms of safety; hence, there were fewer early complications in this study. Moreover, there have been reports of reduced long-term complications of LS, such as adhesions and incisional hernia [40, 41]. The findings of this study suggest that the application of OS in older patients who could have been successfully treated with minimally invasive surgery can lead to serious functional decline. OS not only reduces the patient's QOL but also increases the burden on the family and society and may lead to a waste of medical resources. Combined with previous studies’ results, the superiority of LS would not change even with the conversion from LS to OS, and has greater benefits in the long term. LS in emergency abdominal surgery may not only be safer but also superior in selected patients.

In this study, men were found to be more likely to maintain physical function. This observation may be attributed to factors such as women having lower muscle mass and being at a higher risk of osteoporosis and other bone density loss [42]. It is important to note that the possibility of a type 1 error cannot be ruled out as a statistical limitation. Further research is warranted to delve deeper into gender differences and validate these findings.

This study has some limitations. First, as this was a single-center, retrospective study, we could not determine the possibility of a selection bias. Second, although a multivariate analysis was performed to reduce the influence of confounding factors, this study is a retrospective observational study, and the influence of ASA and type of surgical intervention cannot be completely excluded. Regarding the type of surgical intervention, the overall number of cases were limited, and it was not feasible to analyze the various surgical interventions separately. Although this study was able to report a statistical association between LS and physical function decline, prospective studies are needed to determine the causality. Third, the study did not include factors related to the surgeon. The surgeon's position and experience with LS were not examined in this study. Thus, caution should be exercised when applying our findings to other institutes and countries. Fourth, unmeasured variables, such as previous surgical history, degree of intra-abdominal adhesions, and bowel dilatation, may have contributed to the decision regarding the surgical procedure. In this study, postoperative complications, postoperative ICU period, and intubation period were not considered because the study was examining whether postoperative decline in physical function could be predicted from the surgical procedure. In addition, patients who died during hospitalization were not included in the analysis, which may change the interpretation of the results. Fifth, in this study, similar to previous studies, we defined 65 years and older as elderly. However, there might be significant differences in physiological capacity between 65–70 and 85–90-year-olds. In fact, multivariate analysis showed that age was statistically significant as a factor in the decline of physical function. However, questions remain regarding the definition of elderly. This study showed an association between LS and the maintenance of physical function in older individuals but did not demonstrate a causal relationship. Randomization in an emergency setting may be impractical; however, multicenter studies adjusted for confounding factors are necessary. Besides, although postoperative rehabilitation protocols have been established, there may have been differences in interventions among patients. The possibility that the lifestyle and location after discharge, such as home or a nursing home, may influence functional decline cannot be ruled out. However, early postoperative rehabilitation is also important.

Despite the above limitations, we believe that the findings of this study will provide a firm foundation for future research. The widespread use of laparoscopic approaches could be important for the future of emergency abdominal surgery for individual patients and the healthcare system.

## Conclusions

In emergency abdominal surgery, the choice between LS and OS is associated with postoperative physical functional decline in older individuals. The widespread use of the laparoscopic approach is important for a better future of emergency abdominal surgery, as it provides patient-centered care that maintains the patient's physical function.

### Supplementary Information

**Supplementary Material 1. **

**Supplementary Material 2. **

**Supplementary Material 3. **

## Data Availability

The datasets generated during and/or analysed during the current study are not publicly available, but are available from the corresponding author on reasonable request.

## Abbreviations

ADL: Activities of daily living
Alb: Albumin
ASA: American Society of Anesthesiology
BI: Barthel index
BMI: Body mass index
CCI: Charlson Comorbidity index
CI: Confidence intervals
CRP: C-reactive protein
Hb: Hemoglobin
IQR: Interquartile range
LS: Laparoscopic surgery
OR: Odds ratios
OS: Open surgery
QOL: Quality of life
SSI: Surgical site infection
WBC: White blood cell
