# Supplementary figures and images for: Laparoscopy for emergency abdominal surgery is associated with reduced physical functional decline in older patients: a cohort study
Source: BMC Geriatr. 2024 Mar 12;24:250. doi: 10.1186/s12877-024-04872-y (PMC10936080; doi:10.1186/s12877-024-04872-y)

Additional file 1. Barthel Index to measure the level of independence


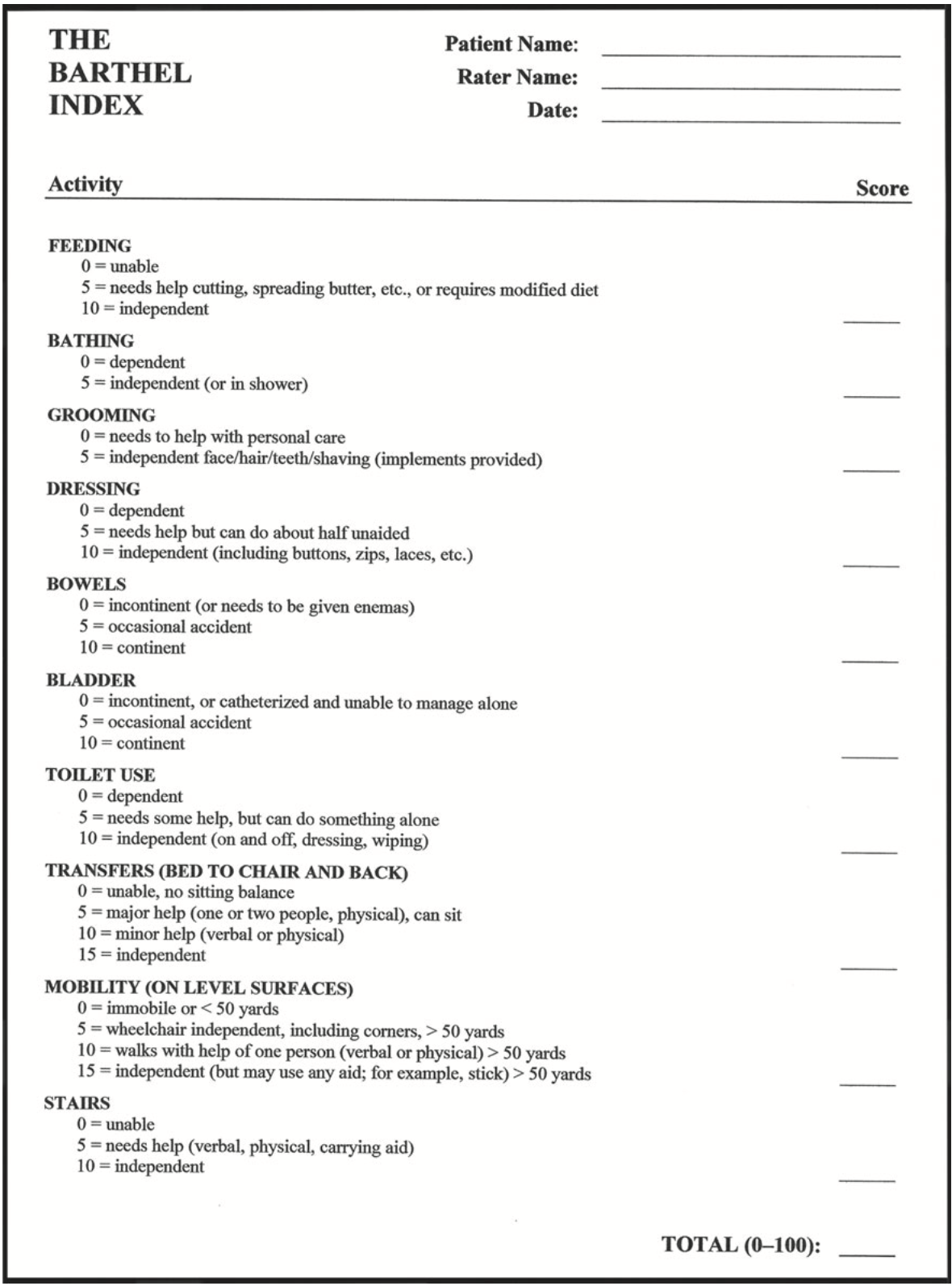

Supplement: Supplementary file 1 — Supplementary Material 1. [file 12877_2024_4872_MOESM1_ESM.docx]
