# Supplementary material for: Laparoscopy for emergency abdominal surgery is associated with reduced physical functional decline in older patients: a cohort study
Source: BMC Geriatr. 2024 Mar 12;24:250. doi: 10.1186/s12877-024-04872-y (PMC10936080; doi:10.1186/s12877-024-04872-y)
Supplement: Supplementary file 2 — Supplementary Material 2. [file 12877_2024_4872_MOESM2_ESM.docx]

Additional file 2. List of the type of surgical intervention

|  | **Laparoscopic**  **Surgey**  **n = 94** | **Open Surgery**  **n = 186** | **Overall** |
| --- | --- | --- | --- |
| Major Surgery | 17 | 106 |  |
| Bowel resection or  Hartmann’s surgery | 7 | 89 | 96 |
| Surgery for diffuse peritonitis | 10 | 13 | 23 |
| Others | 0 | 4 | 4 |
|  |  |  |  |
| Intermediate-Minor | 77 | 80 |  |
| Cholecystectomy | 29 | 15 | 44 |
| Appendicectomy | 24 | 5 | 29 |
| Dissection of adhesions | 14 | 24 | 38 |
| Stoma creation w/o resection | 5 | 7 | 12 |
| Hernia repair | 2 | 18 | 20 |
| Diagnostic LS or OS | 1 | 4 | 5 |
| Others | 2 | 7 | 9 |

Abbreviations: w/o, without; LS, Laparoscopic surgery; OS, Open surgery
