# Supplementary material for: Laparoscopy for emergency abdominal surgery is associated with reduced physical functional decline in older patients: a cohort study
Source: BMC Geriatr. 2024 Mar 12;24:250. doi: 10.1186/s12877-024-04872-y (PMC10936080; doi:10.1186/s12877-024-04872-y)
Supplement: Supplementary file 3 — Supplementary Material 3. [file 12877_2024_4872_MOESM3_ESM.docx]

Additional file 3. Complications stratified by surgical procedure

| Complications, n (%) | **Laparoscopic**  **surgery**  **n = 94** | **Open Surgery**  **n = 186** | **p-value** |
| --- | --- | --- | --- |
| All | 15 (16.0) | 71 (38.2) | <0.001 |
| Major surgery | 17 | 106 | 0.12 |
| CD grade Ⅰ/Ⅱ | 3 (17.6) | 39 (36.8) |  |
| CD grade ≥Ⅲ | 2 (11.8) | 17 (16.0) |  |
| Intermediate-Minor | 77 | 80 | 0.39 |
| CD grade Ⅰ/Ⅱ | 5 (6.5) | 10 (12.5) |  |
| CD grade ≥Ⅲ | 5 (6.5) | 5 (6.3) |  |

Abbreviations: CD, Clavien–Dindo classification
